# Optical pH measurement system using a single fluorescent dye for assessing susceptibility to dental caries

**DOI:** 10.1117/1.JBO.24.1.017001

**Published:** 2019-01-08

**Authors:** Manuja Sharma, Jasmine Y. Graham, Philip A. Walczak, Ryan Nguyen, Lauren K. Lee, Matthew D. Carson, Leonard Y. Nelson, Shwetak N. Patel, Zheng Xu, Eric J. Seibel

**Affiliations:** a University of Washington, Electrical and Computer Engineering, Seattle, Washington, United States; b University of California, Berkeley – University of California, San Francisco, Department of Bioengineering, Berkeley, California, United States; c University of Washington, School of Dentistry, Seattle, Washington, United States; d University of Washington, Department of Microbiology, Seattle, Washington, United States; e University of Washington, Department of Biochemistry, Seattle, Washington, United States; f University of Washington, Human Photonics Lab, Seattle, Washington, United States; g University of Washington, Department of Mechanical Engineering, Seattle, Washington, United States

**Keywords:** fluorescein, anion, dianion, fluorescence, unmixing, caries, pH, plaque, Stephan curve

## Abstract

Sugar-rich diets and poor dental hygiene promote the formation of a biofilm (plaque) that strongly adheres to the dental enamel surface and fosters the evolution of aciduric bacteria. The acid contributes to demineralization of the exterior tooth enamel, which accelerates after the pH drops below a critical value (∼5.5) for extended time periods resulting in the need for restorative procedures. Preventative techniques to alert the dentist and caries-susceptible patients regarding vulnerability to dental decay require a clinical measure of plaque activity. Therefore, there is a need to evaluate the acid production capability of plaque deposits in the pits and fissures of occlusal and interproximal regions. A ratiometric fluorescence pH-sensing device has been developed using an FDA-approved dye and LED excitation. Fluorescein spectral profiles were collected using a spectrometer and analyzed with a spectral unmixing algorithm for calibration over the pH range of 4.5 to 7. An *in vivo* pilot study on human subjects was performed using a sucrose rinse to accelerate bacterial metabolism and to measure the time-dependent drop in pH. The optical system is relatively immune to confounding factors such as photobleaching, dye concentration, and variation in excitation intensity associated with earlier dye-based pH measurement techniques.

## 1 Introduction

Dental plaque formation occurs in a systematic manner, with early bacterial colonizers creating a favorable environment for secondary colonizers.^1^ In the transition from early colonizers to secondary colonizers, the dental plaque microbiome shifts to include a complex mixture of acidogenic (acid-producers) and aciduric (acid-tolerant) species.^1^^–^^3^ Plaque bacterial metabolism of dietary sugar and carbohydrates^4^^,^^5^ produces lactic and other organic acids as by-products. The pH of dental plaques drop following a sucrose rinse and is restored back to baseline pH (now known as the Stephan curve), and repeated time spent below critical pH (∼5.5)^6^ leads to demineralization of enamel.^7^ Organic acids produced by these bacteria, specifically lactic and acetic acid, have been shown to cause enamel demineralization.^3^^,^^4^ This demineralization may progress to the clinical diagnosis of dental caries or breakdown of the tooth enamel surface.^8^

As the plaque deposits mature and increase in thickness, the proportion of organic acid-producing bacterial species within the biofilm steadily increases.^9^ The acid-producing bacterial colonies tend to be anaerobic and therefore the biofilm layer adjacent to the enamel surface is exposed to the most acidic environment.^10^ In addition, the chemical composition of the extracellular polysaccharide matrix surrounding the bacterial colonies was shown to influence the rate of diffusion of hydronium ions through the biofilm, which may render occlusal fissures susceptible to cariogenic attack by increasing the matrix porosity and diffusion of acid into restricted locations.^11^ Below critical pH, the equilibrium^12^ between enamel demineralization and remineralization is acutely disrupted and early stage caries (white spot) lesions begin to form. For a short time, this intact surface layer^13^ hides the deeper (50 to 100  μm) enamel demineralization. Unless the demineralization process is arrested, the outer surface layer degrades, and a visible opening appears in the enamel surface. If this lesion is detected early, interventions such as fluoride varnish application, etc. can prevent further degradation.^14^ Depending on progression of caries through dentin, more advanced lesions may require invasive restorations to repair structural loss.

A study conducted by Stephan^7^ demonstrated the sudden pH reduction following a glucose rinse and longer-term recovery to near-neutral pH. Stephan found that subjects with a high propensity for caries exhibited the lowest pH values following the sugar rinse and required much longer recovery times. Subsequent studies with children and adolescents, using the same sugar rinse approach, yielded a peak and valley Stephan-like time-dependent pH behavior.^15^^–^^17^ However, the pediatric studies differed in their analysis regarding the correlation of caries incidence to the peak and valley pH level.

Historically, electrical probe devices have been used to measure the acidic content of a plaque biofilm.^7^^,^^16^^,^^18^ However, the contact pH sensor may disrupt the plaque and the readings may not be representative of the acidity of the biofilm that is directly attached to the enamel surface. Depending on the plaque age and nutrients available, the biofilm thickness varies over a wide range from 30  μm to over 200  μm.^19^^,^^20^ Furthermore, typical electrical probes are fragile and require frequent calibration. pH indicator strips, aka Litmus papers, have also been used to gauge plaque acidity in a sugar challenge study.^21^ However, pH paper cannot be inserted into the restricted pits and fissures of occlusal surfaces, and the tight interproximal areas between teeth are also challenging. The colorimetric readings of the litmus papers may also be subject to misinterpretation and surface absorbed color-change dyes can leach into the saliva and may result in erratic results. Flexible plastic test strips based on poly(ionic) liquids that anchor the color-sensitive dyes have been developed^22^ but any increase in thickness of these pH strips would severely limit access interproximally and the occlusal pits and fissures would be unreachable. Confocal laser scanning microscopy (CLSM) has been used to measure the pH depth profiles of dental bacteria biofilms.^19^^,^^23^ CLSM investigations provide a valuable insight into the three-dimensional (3-D) structure of plaque-like biofilms, but adapting this technology to the dental clinic is currently not feasible.

This work explores a new technique of measuring biofilm pH that can be implemented *in vivo* using properties of an FDA-approved dye, fluorescein (FL). It is an attractive fluorescent dye that offers high quantum efficiency and is often selected for intensity-based pH sensing. In addition, FL resides predominantly in the extracellular space (exclusion dye)^19^^,^^24^ since it does not penetrate the negatively charged bacterial cell wall. An excitation wavelength near the peak absorption band (∼490  nm) is usually employed and the emission intensity near 520 nm is recorded. FL emission intensity decreases in a nearly linear fashion as the pH is reduced. Unfortunately, fluorescence intensity depends upon several factors including the stability of the excitation light source(s), light scattering, dye photobleaching, dye quenching, etc. Overlooked in adapting FL fluorescence to pH sensing is the change in the relative proportion of its dianion and anion pH-sensitive molecular variants.^25^^,^^26^ As the acidity level increases the predominant fluorescent species shifts from dianion to anion. These two species have overlapping absorption and emission spectral characteristics. Unmixing^27^^,^^28^ of the overlapping spectral emission data using least-square fitting of the endmember dianion and anion fluorescent species is performed to determine the pH. In addition, a judicious choice of excitation wavelength (420 nm) makes it possible to balance the stronger dianion absorption/emission with the weaker anion absorption/emission to optimize the performance of the unmixing algorithm. Other unwanted noise contributions, such as background light and autofluorescence (AF) in the range of 450 to 650 nm, are removed before calculating the biofilm pH.

Therefore, using the spectral unmixing algorithm in combination with the FL fluorescent dye, we achieved many of the desirable features of a ratiometric dental pH sensor such as operation with a single excitation wavelength, noncontact, and immunity from changes in dye concentration, excitation intensity, and photobleaching. A fiber optic probe was developed that can measure acidity levels in difficult to access dental locations such as occlusal pits and fissures. Details of the construction of the fiber optic pH probe, calibration methods, spectral unmixing algorithm, and first-in-human pilot study are presented.

## 2 Methods and Materials

### 2.1 Chemistry

#### 2.1.1 Preparation of fluorescein solutions

1-M stock solution of sodium FL (Sigma Aldrich and ScienceLab) was prepared in deionized water that was diluted to make FL solutions. Buffered FL solutions were used for calibration of the pH device with a conventional pH meter (ThermoFisher Scientific). FL was diluted in phosphate citrate buffer (0.2-M dibasic sodium phosphate, 0.1-M citric acid, pH indicated for each experiment), 0.1-M sodium bicarbonate buffer, or chemically defined medium (CDM) buffer.^29^ FL solutions were stored in test tubes and wrapped in aluminum foil and handled with minimal light exposure. We used different concentrations of the FL solutions ranging from 100 to 600  μM.

### 2.2 Hardware

**Fig. 1:**
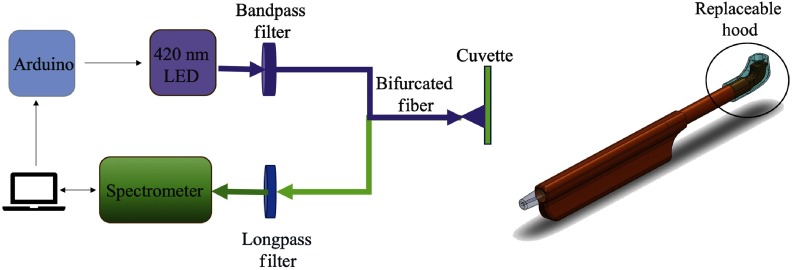
(a) Diagram of electro-optical device and (b) dental probe.

An Ocean Optics spectrometer (USB 2000+) equipped with Ocean View software recorded the fluorescence spectral profile. A pulsed 420-nm LED housed with a driver (ThorLabs, M420F1) served as the FL excitation source. The pulsed LED and spectrometer were synchronized using an Arduino board controlled via computer. A bandpass filter (Semrock, FF01-425/26-25) centered at a 425-nm limited the bandwidth of the LED emission and also attenuated the yellow-green LED defect emission centered near 550 nm.^30^ A 450-nm long pass filter (Semrock, BLP01-458R-25) blocked the strong blue LED emission from entering the spectrometer and distorting the FL fluorescence spectrum. The 1-in. diameter filters were mounted into an in-line holder (Mightex, SPC-FTH-V1). Dual fiber optic cables were used to collect the fluorescence emission as well transmit the LED excitation. The configuration shown in Fig. 1 was used for calibration with FL buffers in a 1-mm glass cuvette (Firefly, GL) at a distance of 3 mm from the light collection/transmitting fiber optic probe (ThorLabs, BFY200HS02). The custom dental probe was used for *in-vitro* testing of tooth samples and *in vivo* plaque pH measurement. An optical power meter (Newport #1830-C) was used to measure the LED output from the probe tip. Due to losses in the optical fiber cables and connectors the output power was in the range of 40 to 180  μW. 500-μm-diameter plastic fiber optics (Fiberfin, FF-SK-20) in the dental probe device were mounted inside a 3-D printed, nonfluorescent black plastic (Hatchbox PLA) holder. Based on the shapes of conventional dental tools, the probe incorporated a 90-deg turn at the tip to simulate clinical dental tools and had a form factor of a toothbrush. Special care was taken in selecting the materials for the probe to avoid unwanted AF. A food-grade silicone molded hood covered the probe tip to reduce ambient light interference. The silicone was made completely black by adding iron oxide powder, 0.3-μm particle size, in the molding mixture. The hood was replaced *in lieu* of clinical hygiene requirements along with cleaning the probe with disinfectant wipes (Metrex Cavicide). In addition, a disposable plastic sheath was used to cover the probe with openings for the fiber optics.

### 2.3 Algorithm

#### 2.3.1 Calibration curve: linear unmixing

**Fig. 2:**
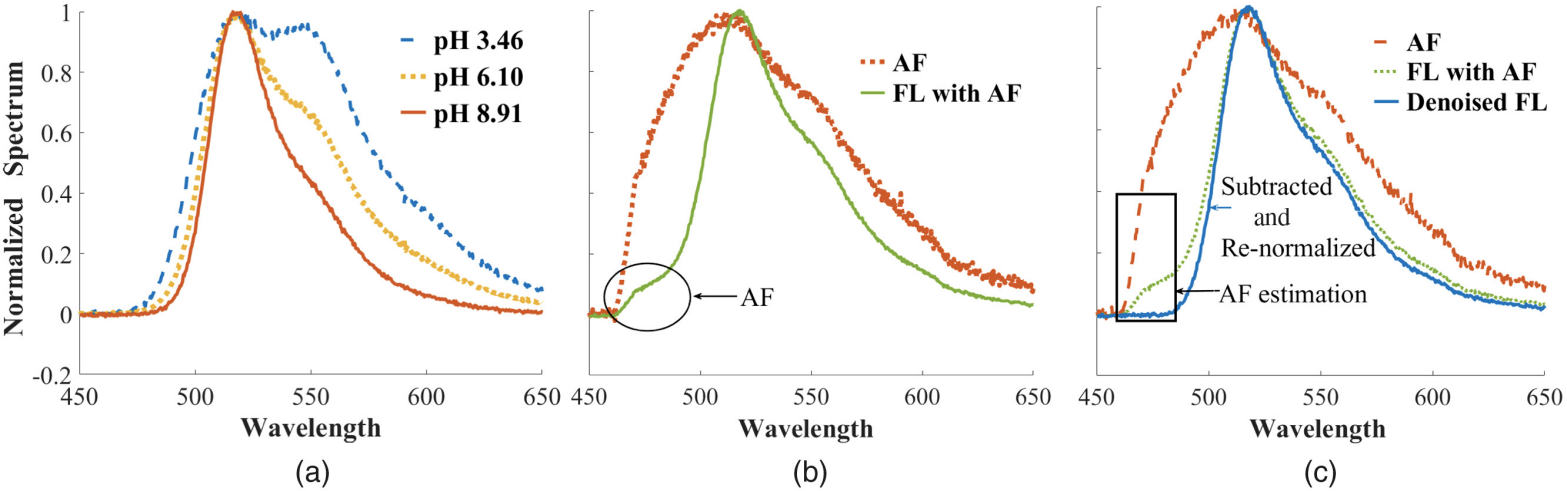
(a) FL emission spectrum (450 to 650 nm) for different pH solutions. (b) Tooth AF using 420-nm LED and FL spectrum with noisy AF (c) AF removal. All signals have been normalized using the peak intensity recorded.

In aqueous solution FL exhibits an equilibrium mixture of four different species (cation, neutral, anion, and dianion) that depends upon pH. Only the dianion and anion species are fluorescent.^25^ With increasing pH of an FL solution, the anion concentration decreases and the dianion concentration increases. For example, at a pH 4, an FL solution will consist of predominantly anions (A) and at a pH 9, the solution will contain mainly dianions (D) resulting in different spectral properties in the 450- to 650-nm range [Fig. 2(a)]. Solutions between pH 4.5 and 7 contain both dianion and anion species resulting in a fluorescent spectral profile that is a mixture of individual emission profiles. Using the spectral properties of endmembers (pH 4 and 9) for pure anion and dianion species, we performed a linear unmixing of the normalized spectral profiles (S) for unknown pH solutions in the 4.5- to 7-range as

$$S_{\mathrm{pH}\_\text{unknown}}=D*S_{\mathrm{pH}9}+A*S_{\mathrm{pH}4}.$$ (1)

The relative proportion of anions (A) and dianions (D) in the solution was obtained using a least mean square (LMS) regression. Using nine samples between pH 4.5 and 7, we obtained two pH calibration curves, one for the dianion fraction in the solution and other for the anion fraction as described in Sec. 3. For this analysis, the recorded spectrum was normalized using the peak intensity value in the spectrum range.

#### 2.3.2 Mitigation of tooth AF

Tooth enamel exhibits a broad fluorescence starting at 450 nm and extending to 700 nm with a peak around 500 nm.^25^ A blue 420-nm LED will excite this AF and contribute a noise source to the FL emission profile as shown in Fig. 2(b). To remove this AF interference, we first measured the AF of the tooth before measuring the FL emission. We then subtracted the AF from the total FL spectrum using a curve fitting algorithm and assumed that the AF was the predominant emission feature in the 460- to 480-nm range. Hence, we scaled the entire AF patient tooth profile with the limited spectral signal using the LMS function to obtain the overall AF spectral contribution. The corrected FL emission (Denoised FL) profile was then renormalized as shown in Fig. 2(c).

#### 2.3.3 Determination of plaque pH

**Fig. 3:**
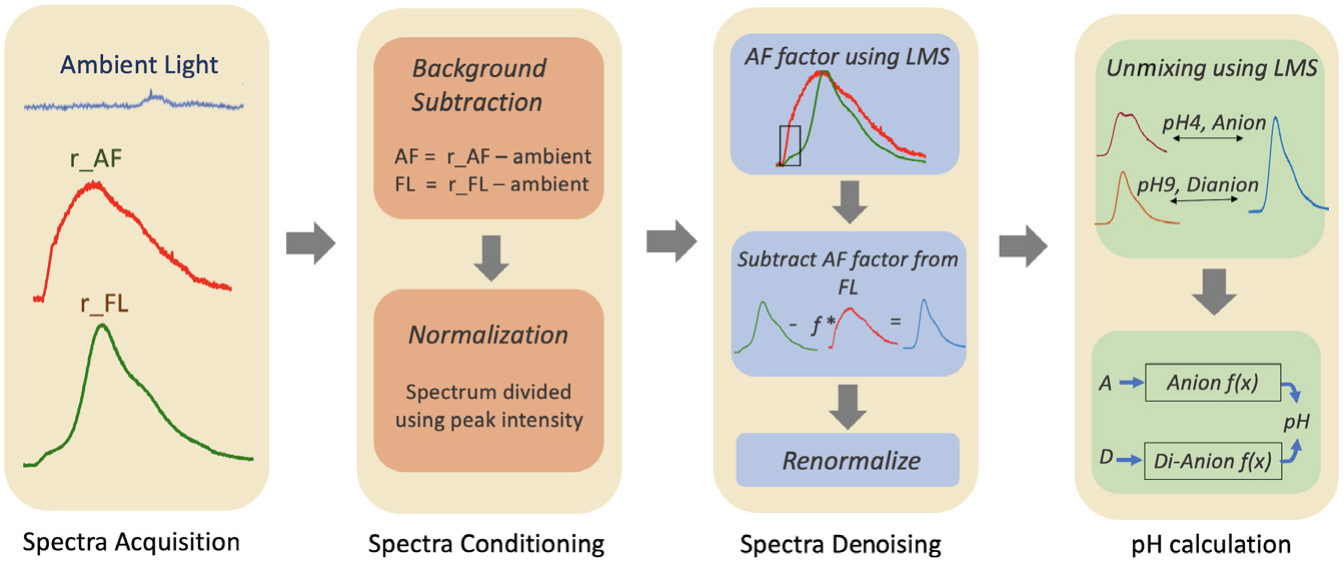
Procedure for plaque pH measurement.

Plaque pH was measured using the FL fluorescence methodology for *in vivo* pilot study. Fluorescence data were collected and processed as described above and summarized in Fig. 3. Ambient light spectrum was collected in addition to AF and FL spectra to perform background light subtraction.

## 3 Experiments and Results

### 3.1 Calibration

**Fig. 4:**
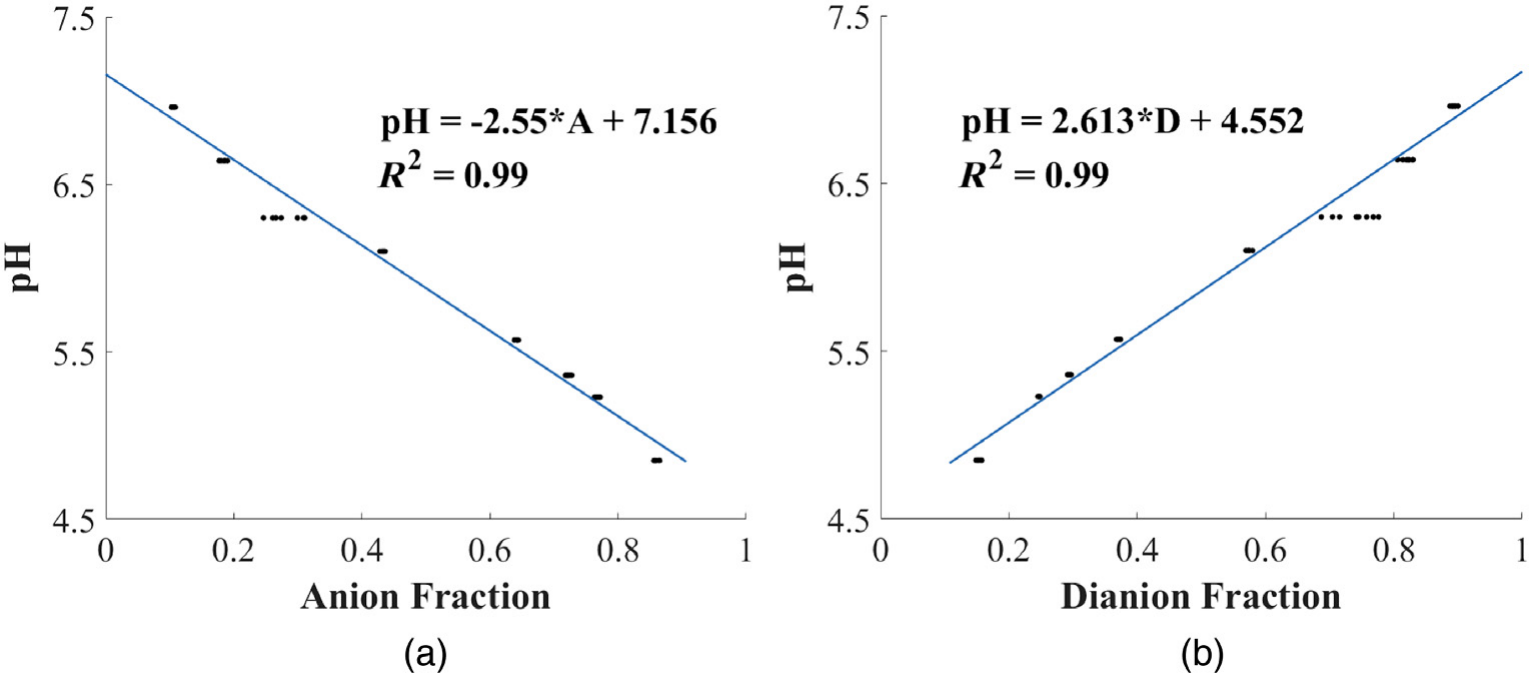
Calibration curves obtained using 200-μM buffered FL solution in 1-mm glass cuvette. (a) Anion calibration curve and (b) dianion calibration curve.

Using a 1-mm glass cuvette and the bifurcated fiber optic positioned 3 mm from the cuvette [Fig. 1(a)], we measured nine different 200-μM FL buffers from pH 4.5 to 7. Each measurement was repeated 10 times to obtain the anion and dianion calibration curves using linear unmixing as shown in Fig. 4. Each of the calibration equations were used to predict pH in the range of 4.5 to 7.

### 3.2 Verification

#### 3.2.1 Buffer samples

**Fig. 5:**
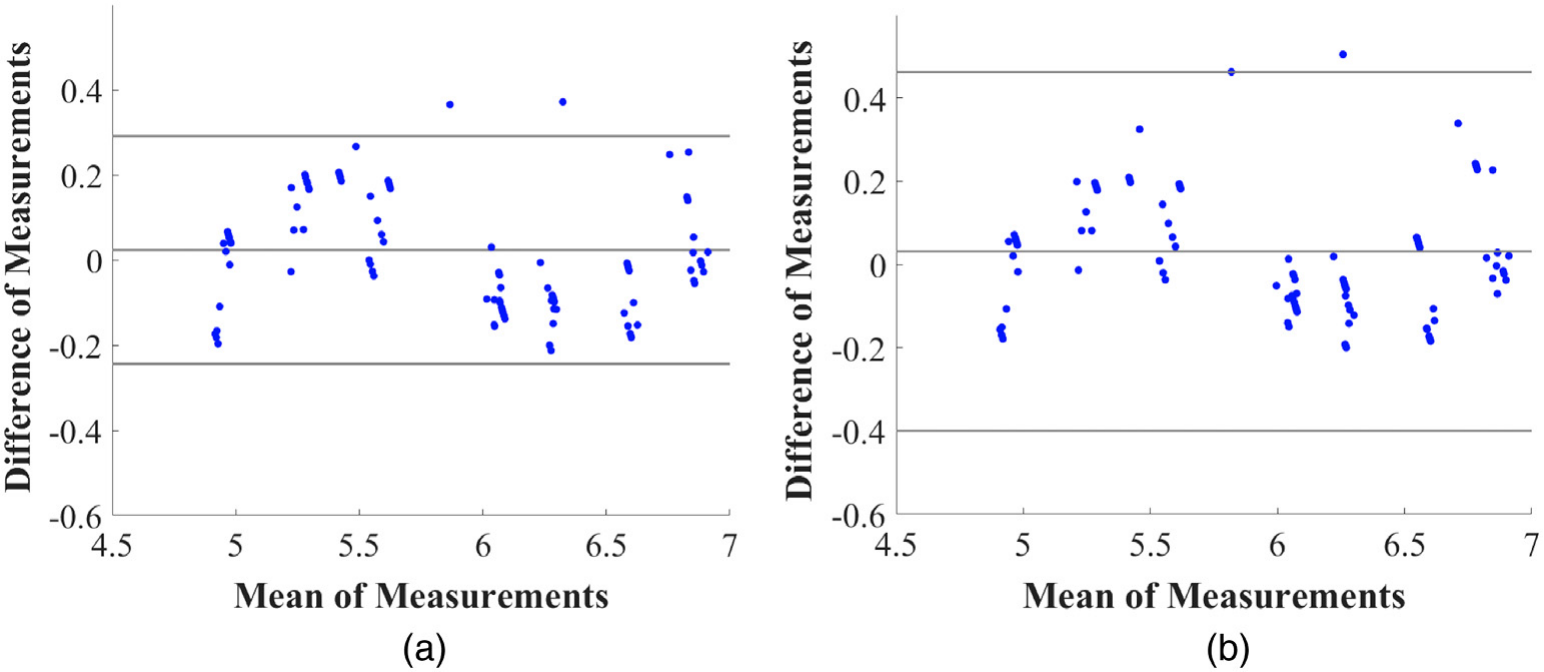
Bland–Altman plot (with 95% agreement) between reference pH meter and predicted pH values using FL for (a) anion and (b) dianion.

We created a test set consisting of different buffer values than those used for calibration in the same pH range. We also varied the concentration of FL from 100 to 600  μM solution and the measurement distance of the cuvette and bifurcated fiber ranging from 1 to 3 mm. Figure 5 shows a Bland Altman plot using 95% limit of agreement between the value measured with the gold standard pH meter and derived using our FL method [Eq. (1)]. Using the anion calibration curve, the root mean square error (RMSE) of 100 samples is 0.14 pH and one standard deviation (SD) is 0.14 pH. By comparison, using the dianion calibration curve the RMSE is 0.2 pH and SD is 0.15 pH.

#### 3.2.2 In vitro tooth samples

**Fig. 6:**
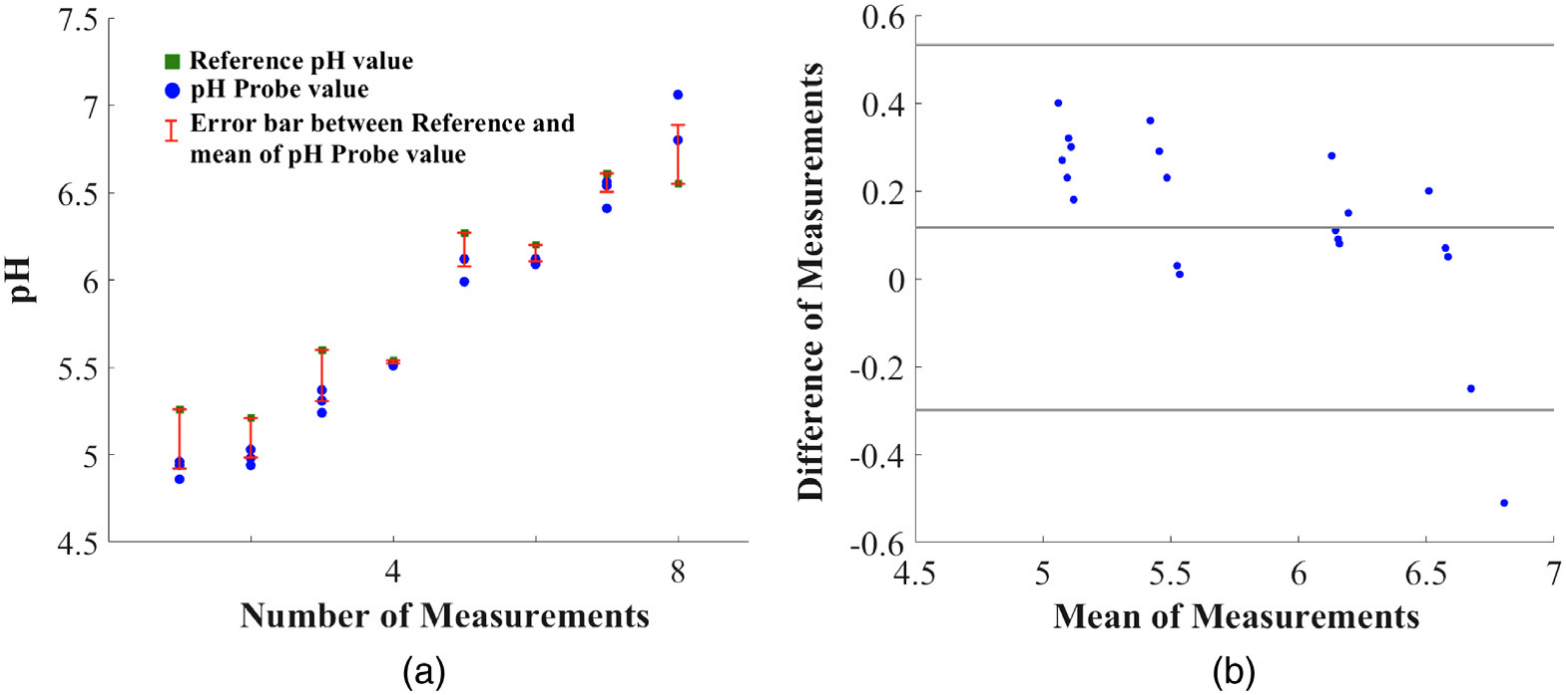
(a) *In vitro* measurements: reference measurements using the conventional pH meter in green square, repeatable measurements using the FL system in blue circles, and error bars between average of the three repeated measurements and reference system in red. (b) Bland–Altman plot between reference pH meter and predicted pH values for *in-vitro* tooth samples (using 95% agreement between variables).

To confirm that AF can be removed from the FL spectrum to predict pH values, we tested buffered FL for different pH values on two extracted molars. Surgically extracted (January 2018) impacted third molars were obtained for *in vitro* study. Samples were refrigerated in 1% Thymol and washed with distilled water before testing. We first recorded the ambient background and AF spectra and then applied a 10-μL drop of buffered FL to the tooth surface (occlusal) and recorded the resultant spectrum. All the measurements with the dental probe [Fig. 1(a)] were taken with a separation distance of 2 mm between the tip of the probe and the FL drop. Teeth were rinsed using water between measurements. The result using the anion calibration curve for two extracted teeth is represented in Fig. 6, we used 200-μM FL for testing. Three measurements were recorded for each buffer value. RMSE and one SD for 24 samples were 0.3 and 0.2 pH, respectively.

### 3.3 Pilot Study: Resting ph and Sucrose Response

**Fig. 7:**
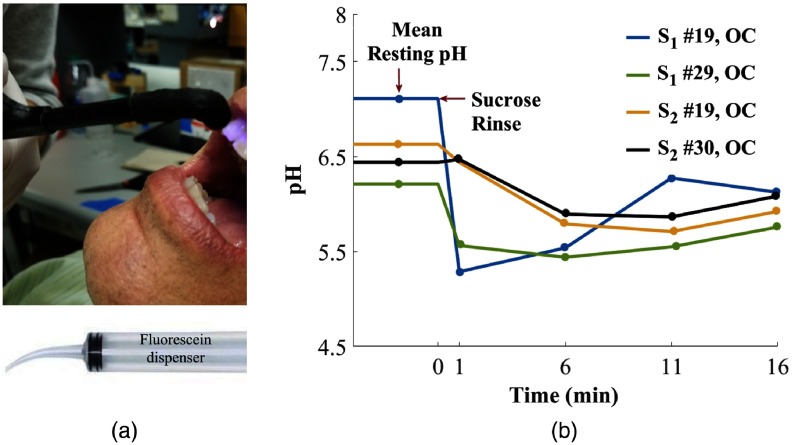
(a) *In vivo* measurement and (b) mean resting pH and sucrose response of two subjects after 0.3-M sucrose rinse. pH value before 0 min indicates mean resting pH of subject’s tooth. Tooth number is listed in the legend along with the surface (OC: occlusal). The time from 1 to 16 min is the dynamic sucrose response obtained by taking measurements every 5 min.

To demonstrate the clinical utility of the FL-based optical pH system, we measured the resting pH (without sucrose) and then monitored the pH response of a 0.3-M sucrose rinse in eight subjects. An equal number of male and female subjects participated in the study with the median age of 23.5 years and mean age of 28 years. Two of the eight subjects voluntarily skipped brushing the day of the study. For testing, a dentist selected one tooth surface in each of the four different quadrants. She used visual inspection to select surfaces with high plaque load while avoiding interproximal spots. For the resting pH, we recorded three spectra: ambient light, AF, and FL applied to the tooth. Each of the readings was repeated twice to measure repeatability. FL (400  μM) was applied using an irrigation syringe like the one shown in Fig. 7.

Subjects then rinsed their mouth to remove any FL and then rinsed and retained 10 ml of 0.3-M sucrose solution for 15 s.^17^ A minute after the sucrose rinse, we measured the first pH value (1 min) and then took three more measurements of selected tooth surface every 5 min. As a control, buccal surface of tooth number 8 was selected with the assumption of low plaque load. Both resting and sucrose drop pH values were measured on the surface. After all the measurements, subjects’ teeth were cleaned by a hygienist and evaluated by a dentist for low/medium/high risk using CAMBRA (Caries Management by Risk Assessment). We had one high risk, one medium risk, and six low risk patients. All human subject involvement was approved under our University of Washington’s IRB (IRB ID STUDY0004940).

Overall resting pH of 36 readings had one SD of 0.25 pH, four samples were removed due to absence of FL spectra reading. The average resting pH on the control tooth was 6.91 and average pH drop was of 0.4 pH after the sucrose rinse. In six out of eight patients, we obtained pH drop of 0.4 pH or more, within 5 min of the sucrose rinse, for at least one quadrant. Sucrose-induced pH response for the lower quadrants for the two subjects who skipped brushing are represented in Fig. 7. Occlusal (OC) surface was used for the results represented. pH region before 0 min indicates mean resting pH of the selected tooth. The time from 1 to 16 min is the dynamic sucrose response obtained by taking measurements every five minutes. The pH drop represented is more than 0.4 pH for the four measurements depicted with a recovery trend toward the resting pH. Lack of a larger, more significant number of patients prohibited a differentiation between low, medium, and high-risk patients on the basis of pH drop or resting pH.

## 4 Discussion

The present device is able to track pH using only one fluorescent dye between 4.5 and 7, ideal for measuring both resting and sucrose-induced plaque pH drop. The 420-nm LED was selected as the excitation source because its emission is absorbed by both the FL anion and dianion species. Furthermore, the two species exhibit distinctive spectral features that can be used for calibration and quantitative measurement of pH. Verification results for the buffer solutions demonstrate that the system can measure pH values over a range of FL concentration. The unmixing algorithm uses normalized spectral data to derive ratiometric fractions of the anion and dianion species. This approach removes changes in the absolute amount of FL, which may be sensitive to photobleaching. Hence, the system is robust since the calibration only depends on the FL spectral band shapes.

Tests of buffered FL on extracted teeth demonstrate that the system can track the pH of FL on teeth with repeatable results, although the AF interference reduces the predicted accuracy in comparison to measurements taken in cuvette. Resting and sucrose rinse data *in vivo* indicate that the device has the capability to measure plaque pH in difficult to access regions such as occlusal pits and fissures. The optical probe design incorporates a replaceable hood that can either be disposed or sterilized between patient examinations. Replacing the 3-D-printed plastic with a smooth surfaced metal or injection molded plastic will further simplify cleaning of the device.

The pilot study highlighted further device improvements required for routine clinical use. The presence of ambient lighting and AF makes the pH measurement location dependent. The repeatability of locating the probe position between the AF, resting pH, and sugar rinse measurement steps makes it difficult to mitigate the AF and ambient light noise. In an improved version of the device, a lock-in amplifier will be added to decrease interference of ambient light. Furthermore, a dye-in-polymer filter that selectively attenuates the ambient fluorescent light from 450 to 650 nm will be added to the probe. Though the AF interference was reduced by using higher FL concentration for *in vivo* testing, the spectral noise removal was susceptible to variation of illumination area within each measurement. This uncertainty can be alleviated by averaging multiple spots on a tooth surface or incorporating an imaging feature that will guide the probe. Other signal processing techniques such as machine learning can be added to improve accuracy and generate a heat map of plaque pH.

The pilot study with low variability in measurement of resting pH shows potential of measuring *in vivo* pH values. The sucrose response shows that the device is capable of measuring *in vivo* the change of pH as a function of time. A longer-term clinical study is needed to determine whether a correlation can be established between susceptibility of caries and the time profile of the sucrose challenge. With only one high risk subject and absence of patients with active caries, it was difficult to draw any correlation between pH and caries detection. Also, most of the subjects had good dental hygiene, which lowered the accumulation of plaque, hindering the device functionality. The present study is also limited by the lack of a gold standard for measuring intraoral pH. The pH microelectrode or even pH strips cannot be used on the irregular pits and fissures of occlusal surfaces. In future studies, we can measure the pH of scraped plaque of one location per patient using a conventional pH meter to compare with the optical method. However, the scraped plaque will be diluted, disturbed, and prone to sampling error—all of which could affect the pH measurement. In addition, in this study, the FL was not sufficiently viscous to stick to interproximal areas making it difficult to measure pH at those locations. Measurement of pH in the maxillary arch was facilitated by the use of a dental chair otherwise those locations would also suffer from lack of retention of FL. This retention of the FL solution can be improved by adding glycerol to the solution. In future studies, better control selection can be achieved by explicitly noting surfaces with lower plaque rather than using the same control for all the subjects.

A low-cost clinical device may require replacement of the spectrometer with multiple filtered photodiodes sensitive to selected FL emission wavelengths (e.g., 520 and 560 nm). A similar FL intensity-based design was presented in a past work^31^ and can be modified to include only one LED excitation source and multiple filtered detectors or a detector array. With LED and photodiode components, the system could run on an embedded platform providing easier implementation of lock-in amplification and opportunity to develop a better user interface.

## 5 Conclusion

The noncontact, optical pH measurement system enables testing of plaque metabolism and is independent of specific species of dental biofilm. Furthermore, the device uses an FDA-approved FL dye that has been used by ophthalmologists at concentration levels over hundred times greater^32^ than our proposed method. The compact fiber optic-based device can be used to probe difficult to access occlusal and interproximal locations, paving the way for better understanding the correlation between plaque metabolic acidity and caries susceptibility.
