# Optical pH measurement system using a single fluorescent dye for assessing susceptibility to dental caries (Erratum)

**DOI:** 10.1117/1.JBO.26.1.019801

**Published:** 2021-01-13

**Authors:** Manuja Sharma, Jasmine Y. Graham, Philip A. Walczak, Ryan Nguyen, Lauren K. Lee, Matthew D. Carson, Leonard Y. Nelson, Shwetak N. Patel, Zheng Xu, Eric J. Seibel

**Affiliations:** a University of Washington, Electrical and Computer Engineering, Seattle, Washington, United States; b University of California, Berkeley—University of California, San Francisco, Department of Bioengineering, Berkeley, California, United States; c University of Washington, School of Dentistry, Seattle, Washington, United States; d University of Washington, Department of Microbiology, Seattle, Washington, United States; e University of Washington, Department of Biochemistry, Seattle, Washington, United States; f University of Washington, Human Photonics Lab, Seattle, Washington, United States; g University of Washington, Department of Mechanical Engineering, Seattle, Washington, United States

## Abstract

The erratum corrects a grant number listed in Acknowledgments section of the original article.

This article [*J. Biomed. Opt.* **24**(1), 017001 (2019) doi: 10.1117/1.JBO.24.1.017001] was originally published on 8 January 2019 with an erroneous grant number in the Acknowledgments section. The original grant listed was NSF PFI: BIC 11631146; the correct grant number is NSF PFI: BIC 1631146.

The error was corrected on 31 December 2020.

